# MicroRNA-409-3p promotes osteoblastic differentiation via activation of Wnt/β-catenin signaling pathway by targeting *SCAI*

**DOI:** 10.1042/BSR20201902

**Published:** 2021-01-07

**Authors:** Nan Chen, Hao Yang, Lijun Song, Hua Li, Yi Liu, Di Wu

**Affiliations:** 1 Orthopedics Department, First Affiliated Hospital of Kunming Medical University, Kunming 650032, Yunnan Province, China; 2 Department of Geriatric Medicine, First Affiliated Hospital of Kunming Medical University, Kunming 650032, Yunnan Province, China

**Keywords:** miR-409-3p, osteoblast differentiation, SCAI, Wnt/β-catenin

## Abstract

Osteogenic differentiation is an important process of new bone formation, microRNA-409-3p (miR-409-3p) has been reported to be up-regulated in the osteogenic differentiation of human bone marrow mesenchymal stem cells (MSCs). The present study aimed to investigate the regulatory effect of miR-409-3p on osteogenic differentiation of MSCs and its molecular mechanism. The expression of miR-409-3p in osteoblast (human skull osteoblast, HCO) and bone marrow-derived MSCs (MSC-A, MSC-B, MSC-U) were detected by reverse transcription-quantitative polymerase chain reaction (RT-qPCR). The binding of miR-409-3p to suppressor of cancer cell invasion (*SCAI*) in MSC-B was investigated by performing a dual-luciferase reporter gene assay. MSC-B was selected to transfect with miR-409-3p analog/complementary sequence (cs), miR-409-3p analog + SCAI and miR-409-3p cs + small interfering (si)-SCAI, as well as control, respectively. The alkaline phosphatase (ALP) activity, Alizarin Red staining, and the expression of osteogenic markers (ALP, osteocalcin (OCN), osteopontin (OPN), runt-related transcription factor 2 (RUNX2)) in MSC-B during osteoblastic differentiation were tested by RT-qPCR and Western blotting, respectively. Additionally, the Wnt/β-catenin pathway was inhibited by dickkopf-related protein 1 (DKK-1) to get the roles of miR-409-3p during the osteoblastic differentiation of MSC-B when transfected with miR-409-3p analog. The expression of miR-409-3p in HCO was higher than that in these three MSCs and showed an increasing time-dependent trend on the 0 and 21st day of osteoblastic differentiation. MiR-409-3p directly regulated *SCAI* by targeting *SCAI* 3′UTR. Further, miR-409-3p suppressed *SCAI* expression, but SCAI up-regulation suppressed the osteoblastic differentiation, as well as reduced the relative mRNA/protein expression of Wnt/β-catenin signaling pathway-related genes (Axis inhibition protein 1 (AXIN1), β-catenin, Lymphoid Enhancer Binding Factor 1, Cellular-myelocytomatosis (c-myc) and cyclin D1). Importantly, disruption of Wnt signaling also blocked miR-409-3p induced osteoblastic differentiation of MSCs. Therefore, miR-409-3p promotes osteoblastic differentiation through the activation of the Wnt/β-catenin pathway by down-regulating SCAI expression.

## Introduction

Mesenchymal stem cells (MSCs) extracted from bone marrow can form osteoblasts through osteogenic differentiation, which was first discovered by Friedenstein et al*.* in the late 1960s to early 1970s [1]. Osteoblasts participate in the formation of bone and work together with osteoclasts, which is crucial for the repair of fractures, bone development and maintenance [2]. Abnormal differentiation of these two cells may cause several orthopedic diseases, such as osteoporosis, osteoarthritis and fractures, subsequently affect patients’ physical health and life expectancy [3]. However, the proliferation and differentiation capacity of MSCs is limited [4]. Therefore, strengthening the research on the regulation mechanism of induced stem cell differentiation is helpful for curing diseases.

MicroRNAs (miRNAs) are small and single-stranded RNAs that play important regulatory roles in mammals by pairing with mRNAs of target genes to guide their post-transcriptional inhibition [5], which has no effect on the stability of target mRNA. Their target genes play crucial roles in controlling the migration, differentiation, apoptosis and cell cycle progression of cancer-related cells. In previous studies, a series of miRNAs were also involved in MSCs differentiation. For example, miR-194 [6], miR-96 [7], miR-433-3p [8] promote osteoblastic differentiation, and conversely, miR-340 [9], miR-223 [10], miR-155 [11] inhibit osteoblastic differentiation. MiRNAs regulate signal molecules and transcription factors to a certain extent, and connect with signal pathways to form a regulatory system of osteogenic differentiation [12]. Recently, microRNA-409-3p (miR-409-3p) was demonstrated that apparently up-regulated during the osteogenic differentiation of human bone marrow MSCs [13]. Additionally, Cao et al*.* illustrated that overexpression of miR-409-3p prevented breast cancer cell invasion and might be related to the prognostic treatment of breast cancer patients [14]. However, the specific regulatory mechanism of miR-409-3p during the osteoblast differentiation is not clear. Our research aims to get the role of miR-409-3p on osteoblastic differentiation and the underlying mechanism of this process.

Several signaling pathways are indispensable for the regulation of osteoblastic differentiation such as bone morphogenetic protein (BMP), transforming growth factor-β (TGF-β), Wnt/β-catenin, Notch and Hedgehog [15–18]. Wnt/β-catenin signaling pathway is genetically highly conserved and plays crucial roles in regulating cell proliferation, differentiation and cell fate during embryonic development [19]. Nowadays, the importance of Wnt/β-catenin signaling in miRNA regulation is extensively established. Thus, targeting the Wnt pathway may be a new insight to find the potential mechanism in bone formation. In a previous study, Chen et al*.* [20] reported that down-expression of SCAI (suppressor of cancer cell invasion) promotes glioma cell invasion through Wnt signaling activation, but SCAI overexpression inhibited.

Therefore, our study aimed to investigate the relationship between miR-409-3p and *SCAI* and their roles in the process of osteoblastic differentiation of MSCs, thereby providing the potential strategy to improve the treatment of bone injury diseases.

## Materials and methods

### Cell culture

Human skull osteoblasts (HCOs), MSC-A, MSC-B and MSC-U were purchased from BeNa culture collection (Shanghai, China). Mediums including high-glucose Dulbecco’s Modified Eagle’s Medium (high-DMEM, Invitrogen) and MSC Basal Medium (Gibco, Invitrogen Corporation, Carlsbad, CA, U.S.A.) were used to culture cells, respectively. The osteoblastic phenotypes include alkaline phosphatase (ALP) activity and Alizarin Red staining (ARS), which were detected on the 0, 7th, and 21st days of HCO and osteogenesis-induced MSCs.

### Osteoblastic differentiation

Original α-minimum essential medium (α-MEM) was used to culture MSCs with the condition of 5% CO_2_ at 37°C for 48 h. After approaching confluence, the α-MEM was replaced with osteogenic induction medium, which contained α-MEM and supplemented with 10% FBS, 10 mM β-glycerophosphate, 50 μM l-ascorbic acid and 100 nM dexamethasone were prepared for culturing cells (Sigma–Aldrich, St. Louis, MO, U.S.A.). Osteogenic induction of MSCs was performed at 7 and 21 days, and the medium was changed every 3 days.

### Transfection

Transfected targets including miR-409-3p analog, miR-409-3p complementary sequence (cs), control analog, control complementary sequence and small interfering RNA (siRNA) for *SCAI*, which were prepared by GenePharma Co, Ltd. (Shanghai, China) and QIAGEN (Hilden, Germany), separately. These all sequences are shown in Table 1. Additionally, a concentration level of 35–50 nM of these targets was transfected into MSC-B cells using the Lipofectamine 2000 transfection reagent (Invitrogen).

**Table 1:** Sequences of the transfected targets

Gene	Forward primer (5′–3′)	Reverse primer (5′–3′)
MiR-409-3p analog	GAAUGUUGCUCGGUGAACCCCU	GGGUUCACCGAGCAACAUUCUU
MiR-409-3p cs	AGGGGUUCACCGAGCAACAUUC	AUGUUGCUCGGUGAACCCCUUU
C analog	UUCUCCGAACGUGUCACGUUU	ACGUGACACGUUCGGAGAAUU
C cs	UUCUCCGAACGUGUCACGUUU	ACGUGACACGUUCGGAGAAUU
Si-SCAI	AAUUACCUAUGUCUUAAACAA	GUUUAAGACAUAGGUAAUUUU

Abbreviations: C analog, control analog; C cs, control complementary sequence; miR-409-3p cs, miR-409-3p complementary sequence.

### Reverse transcription-quantitative polymerase chain reaction

In accordance with the manufacturer’s instructions, the TRIzol kit (Sangon Biotech, Shanghai, China) was used to extract total RNA in these cells. Then ultraviolet spectrophotometry and agarose gel electrophoresis were used to detect the concentration and purity of the extracted total RNA. After then, reverse transcription-quantitative polymerase chain reaction (RT-qPCR) was used to detect miR-409-3p and other genes expression, and strictly according to reagent reaction conditions and reaction procedures. All qPCR primers were shown in Table 2. U6 and glyceraldehyde 3-phosphate dehydrogenase; (GAPDH) were considered as internal control for miRNA and mRNA, respectively. The relative expression of these samples in experiments is calculated through the 2^−ΔΔ*C*_t_^ method.

**Table 2:** Primer sequences of the genes for qPCR

Gene	Forward primer (5′–3′)	Reverse primer (5′–3′)
*MiR-409-3p*	GAATGTTGCTCGGTGA	GTGCAGGGTCCGAGGT
*SCAI*	GGTTCTGGTGATAGCAGTCAT	CTGCCACTGCTTCTGTCCATA
*AXIN1*	GAAGACGGCGATCCATCG	GGATGCTCTCAGGGTTCT
*β-catenin*	GCTAGTTGGGTTTCGTCGCC	GCTCCTTGGCGGATACCATC
*LEF1*	CCGAAGAGGAAGGCGATTTAGCT	GCTCCTGAGAGGTTTGTGCTTGTCT
*c-myc*	CTACCAGCAGCAGCAGAGC	CGTCCGGGTCGCAGATGAA
*cyclin D1*	GCGTACCCTGACACCAATCT	GCTCCAGAGACAAGAAACG
*GAPDH*	ACCACAGTCCATGCCATCAC	TCCACCACCCTGTTGCTGTA
*U6*	CTGGTAGGGTGCTCGCTTCGGCAG	CAACTGGTGTCGTGGAGTCGGC

Abbreviations: AXIN1, axis inhibition protein 1; c-myc, cellular-myelocytomatosis; LEF1, lymphocyte enhancer-binding factor 1.

### Western blotting

Radio Immunoprecipitation Assay lysis buffer (Sangon Biotech, Shanghai, China) and phenyl methane sulfonyl fluoride (PMSF) was added to gently lyse cells on ice to obtain the proteins of different samples. All the proteins were isolated by 10% sodium dodecyl sulphate/polyacrylamide gel electrophoresis (SDS/PAGE) and then 100 μg were further transferred to polyvinylidene fluoride (PVDF) membranes sealed in 5% skimmed milk. And proteins were incubated with diluted primary antibodies and secondary antibodies, and then membranes were eluted through the buffer solution of Tris-buffered saline Tween-20. The enhanced ECL chemiluminescence detection kit (cat. no. 36222ES60; Shanghai Yeasen Biotechnology Co., Ltd., Shanghai, China) was used to incubate the membrane so that the blotting membrane is fixed, and the final protein or nucleic acid band was displayed on the X-ray film to achieve the purpose of protein determination.

### ALP and ARS

After culturing for indicated time (0, 7, 21 days), HCO and osteogenic induction of MSCs (1 × 10^6^ cells/well) were washed with PBS and fixed with 4% formaldehyde in 90% ethanol for 30 s at room temperature. Then removing the fixative, and the ALP activity was evaluated with the detection Kit (Yeasen, Shanghai, China) according to the manufacturer’s instructions. Cell lysates were incubated with *p*-nitrophenyl phosphate solution for 30 min (pH 9.8). The corresponding absorbance of extracts at 405 nm was finally measured using a microplate reader. The induction of MSCs was rinsed three times with 1× PBS and further fixed by 1 ml of 95% ethanol at room temperature for 15 min. After air drying, they were covered with ARS solution (1%, pH 4.2) for 5 min. The dye was then removed and rinsed by ddH_2_O. Finally, we took pictures under an inverted fluorescence microscope (Olympus, Japan). Additionally, ARS was performed to observe calcium mineralized level. ARS calcium precipitation was fixed with acetic acid (10%, v/v) and methanol (20%, v/v) and then washed in distilled water for 10 min. The absorbance of the extracts at 540 nm was also determined using a microplate reader.

### Luciferase reporter assay

MiR-409-3p may bind to SCAI 3′UTR. The wild-type SCAI 3′UTR and mutant SCAI 3′UTR DNA sequences were designed and synthesized by GenePharma Co, Ltd. (Shanghai, China). Wild-type and mutant-type SCAI 3′UTR sequences were inserted into the pmirGLO Dual-Luciferase miRNA Target Expression Vector (Promega, E1330), and wild-type and mutant luciferase reporter plasmids were constructed, then the luciferase plasmids to were transfected to MSC-B cells. The diluted plasmid DNA was mixed with miR-409-3p analog/C and the corresponding transfection reagent Lipofectamine 2000 (Invitrogen) at room temperature for 20 min, then add to the culture well. Besides, MSC-B cells were transfected with pcDNA3.1-GFP vector using Lipofectamine 2000 reagent (Invitrogen). After cell transfection for 48 h, fluorescence microscopy was used to detect the transfection efficiency in MSC-B cells, and green fluorescence (expression of green fluorescent protein, GFP) was considered successful. Then luciferase activities at 560 nm after the transfection were further determined by Dual-luciferase Reporter Gene System (Promega, E1910).

### Statistical analysis

GraphPad Prism 6.0 software was used to process data. Student’s *t* test was prepared for statistical analysis. *P*<0.05 was defined as significant difference. All experiments were independently performed three times.

## Results

### MiR-409-3p was up-regulated during osteoblastic differentiation

**Figure 1:**
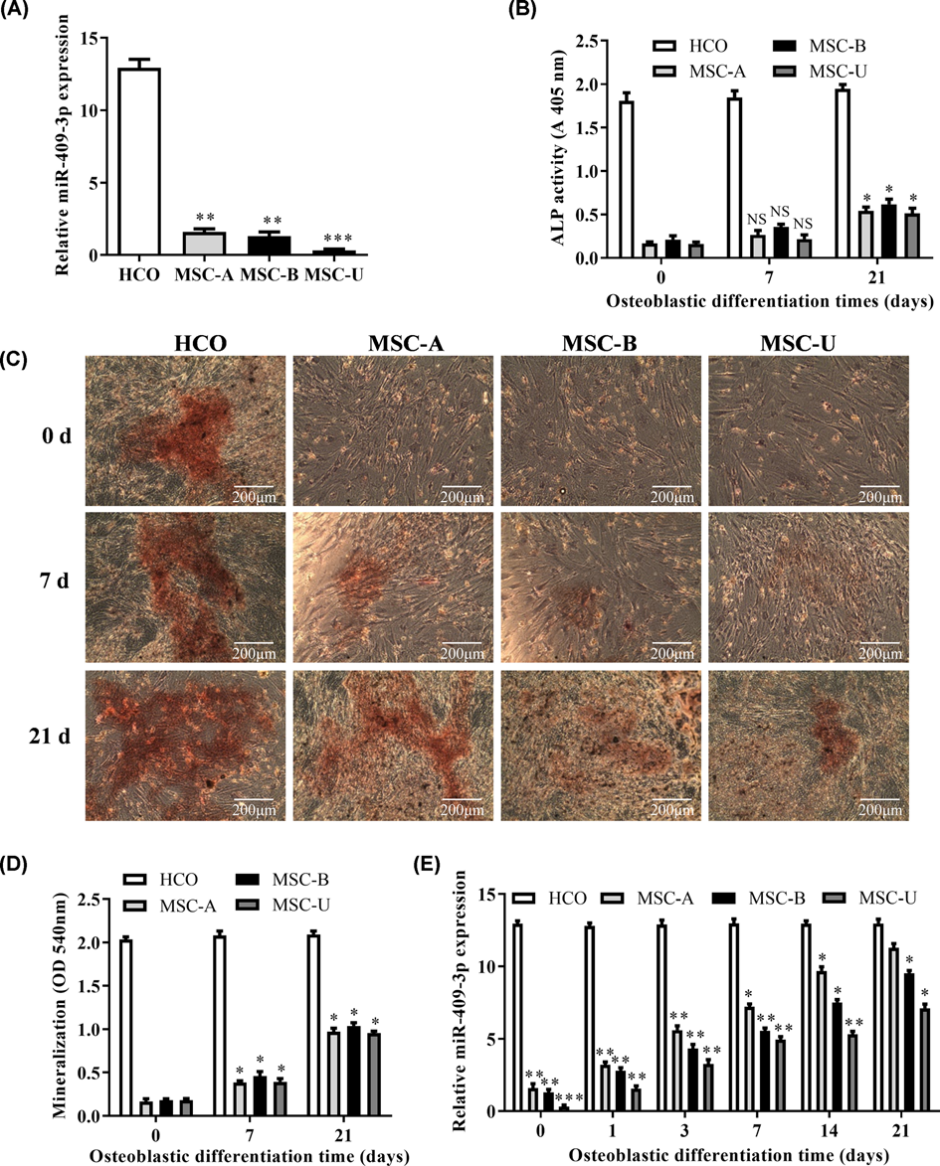
Expression of miR-409-3p in MSCs and HCO as detected by RT-qPCR (**A**) Relative expression of miR-409-3p existed in HCO and MSCs (vs. HCO). (**B**) Determination of ALP activity in HCO and MSCs on days (0, 7, 21) of osteogenic differentiation. (**C,D**) Determination of mineralization in HCO and MSCs on days (0, 7, 21) of osteogenic differentiation. (**E**) Relative expression of miR-409-3p in HCO and MSCs (MSC-A, MSC-B, MSC-C) on days (0, 1, 3, 7, 14, 21) of osteogenic differentiation (vs. on day 0). *P<0.05, **P<0.01, ***P<0.001. Abbreviation: NS, not significant.

After HCO and MSCs cells were cultured to a degree of fusion of 80–90%, we detected the expression of miR-409-3p in HCO, MSC-A, MSC-B, and MSC-U, and found that miR-409-3p expression of MSCs were lower than HCO (Figure 1A, *P*<0.01). Next, MSCs were induced by the osteogenic induction medium, and the results showed that the ALP activity of MSCs continues to increase with the osteogenic differentiation of HCO and MSCs on days 0, 7, 21 (Figure 1B, *P*<0.05), as well as the mineralization nodules in MSCs (Figure 1C,D, *P*<0.05). Meanwhile, miR-409-3p expression was continuously increased in all MSCs, and its expression in MSC-A and MSC-B was higher than in MSC-U. Besides, significant changes occurred in MSC-B in 21 days (Figure 1E, *P*<0.05). Thus, MSC-B was used in the following experiments for finding the potential mechanism of miR-409-3p in osteoblastic differentiation.

### MiR-409-3p enhanced osteoblastic differentiation

**Figure 2:**
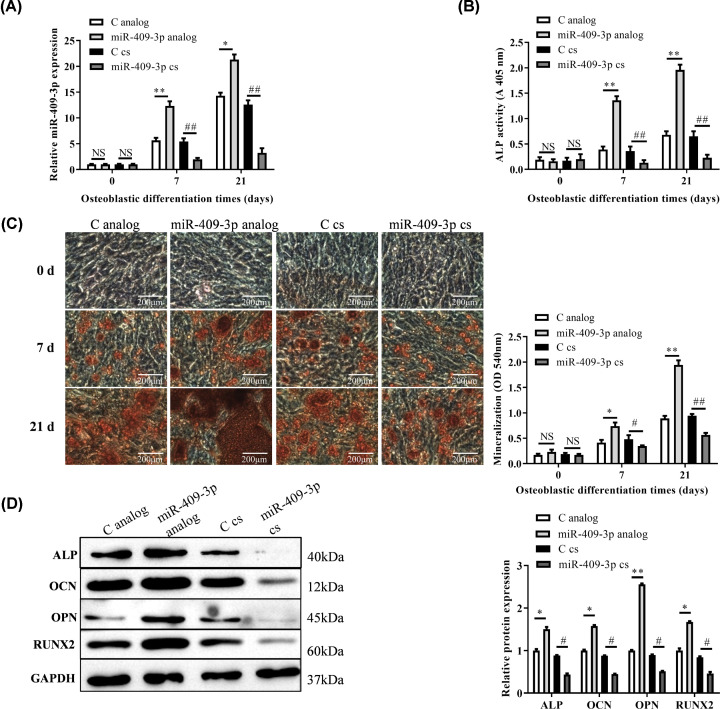
Identification of osteoblastic differentiation (**A**) miR-409-3p expression was identified on 7th and 21st days of osteoblastic differentiation of MSCs. (**B**) ALP activity, (**C**) mineralization nodules in analog and cs groups on days 7 and 21. (**D**) Western blot shown the up/down-expression in above two groups of osteogenic marker proteins (ALP, OCN, OPN, RUNX2) (vs. C analog/cs). */^#^*P*<0.05, **/^##^*P*<0.01 (*, analog group; #, cs group). Abbreviations: C analog, control analog; C cs, control complementary sequence; miR-409-3p cs, miR-409-3p complementary sequence; NS, not significant.

MiR-409-3p analog and cs were transfected into MSC-B cells on days 0, 1, 3, 7, 14, 21 of osteogenesis differentiation, respectively. On the 7th and 21st days, the expression of miR-409-3p analog were increased but miR-409-3p cs decreased (Figure 2A, *P*<0.01, compared with C). Additionally, the ALP activity of miR-409-3p analog group was higher in than the C analog group, but cs group showed an opposite result (Figure 2B, *P*<0.01). Furthermore, the mineralization nodules significantly increased in miR-409-3p mimic while slightly decreased in miR-409-3p cs (Figure 2C, compared with C analog/cs). Meanwhile, osteogenic markers (ALP, osteocalcin (OCN), osteopontin (OPN) and Runt-related transcription factor 2 (RUNX2)) expression were all increased in analog group but decreased in cs group on the 21st day of osteoblastic differentiation of MSCs (Figure 2D, *P*<0.05). These results showed that overexpression of miR-409-3p could promote the osteogenic differentiation of MSC-B cells*.*

### *SCAI* was a target gene of miR-409-3p

**Figure 3:**
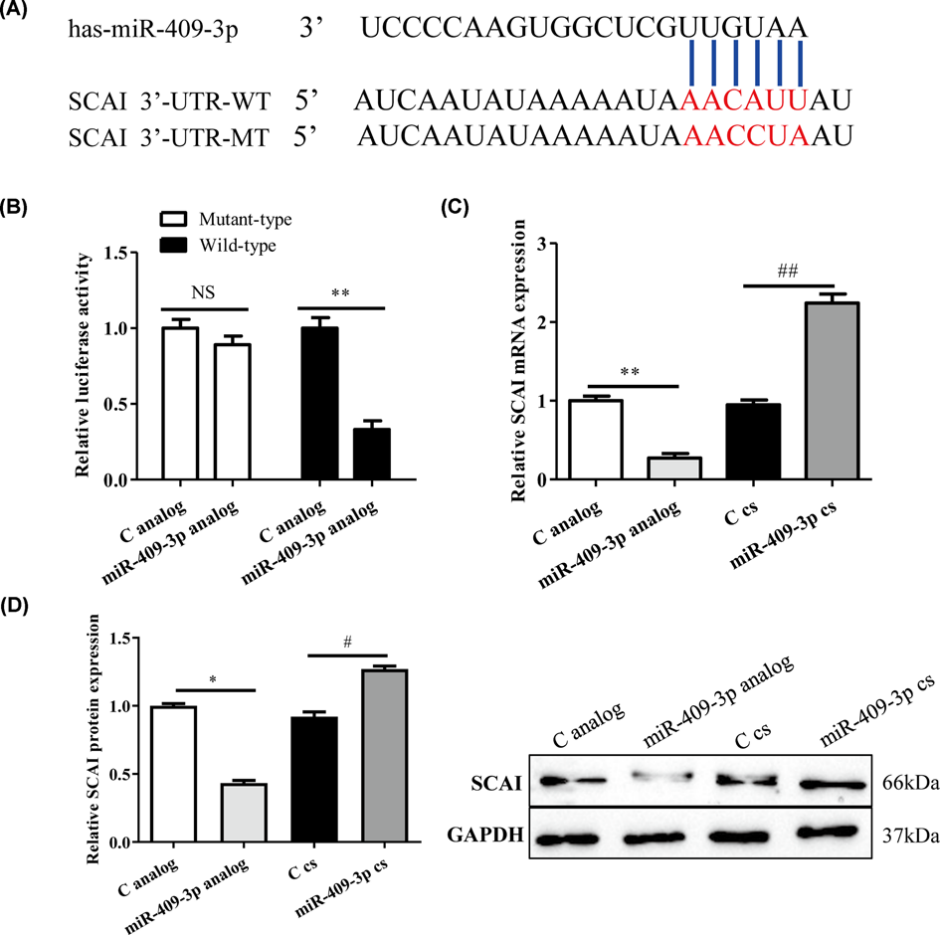
Predicted binding between miR-409-3p and *SCAI* and their expression level (**A**) The binding sites of miR-409-3p in *SCAI* 3′-UTR. (**B**) MiR-409-3p regulated the transcriptional activity of *SCAI* as detected by dual-luciferase reporter gene assay. (**C**) Relative expression of *SCAI* was detected by RT-qPCR in miR-409-3p analog and cs groups (vs. C analog/cs). (**D**) Relative expression of SCAI was detected by Western blotting in miR-409-3p analog and cs groups (vs. C analog/cs). *P<0.05, **P<0.01 (*, analog group; #, cs group). Abbreviations: C analog, control analog; C cs, control complementary sequence; miR-409-3p cs, miR-409-3p complementary sequence; NS, not significant.

We determined the binding site of miR-409-3p and *SCAI* by TargetScan (www.targetscan.org/), then UUGUAA was predicted to be the binding site of miR-409-3p to *SCAI* 3′UTR (Figure 3A). After co-transfection of MSC-B cells, the transfection efficiency was validated by observing GFP in fluorescence microscopy images. As expected, the transfection efficiency reached approximately 90% (Supplementary Figure S1A). In addition, miR-409-3p expression was markedly up-regulated after transfection of the miR-409-3p analog compared with transfection of miR-409-3p control (Supplementary Figure S1B). A luciferase reporter showed that a significant reduction in the luciferase activity after the co-transfection of miR-409-3p analog and *SCAI* wild-type. In contrast, miR-409-3p cs co-transfection with *SCAI* wild-type significantly increased the luciferase activity (Figure 3B, *P*<0.05). However, the luciferase activity of *SCAI* mutant-type combined with miR-409-3p analog/cs showed slight change when compared with C analog/cs. Thus, *SCAI* may be a target for miR-409-3p in osteogenesis differentiation. Next, we studied the correlation between the regulation of miR-409-3p and the expression of target gene SCAI mRNA/protein in osteogenesis differentiation. The miR-409-3p analog and cs showed down- and up-expression in the mRNA/protein level of SCAI, respectively (Figure 3C,D, *P*<0.05). Taken together, miR-409-3p suppressed *SCAI* expression during the osteogenesis differentiation of MSC-B.

### MiR-409-3p suppressed *SCAI* may promote the Wnt/β-catenin pathway

**Figure 4:**
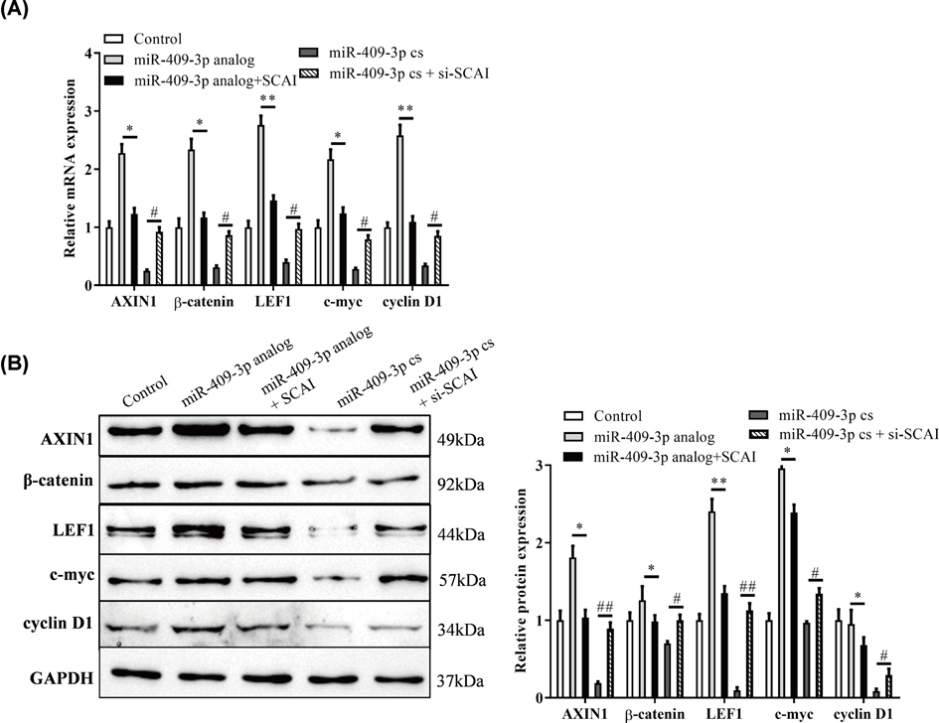
MiR-409-3p activates *SCAI* involved Wnt/β-catenin pathway (**A**) Relative mRNA expression of the Wnt pathway-related genes in transfected osteoblasts using RT-qPCR analysis (vs. Control). (**B**) Relative protein expression of the relevant genes in transfected osteoblasts using Western blot analysis (vs. Control). *P<0.05, **P<0.01 (*, analog group; #, cs group). Abbreviation: miR-409-3p cs, miR-409-3p complementary sequence.

The relative expression of the Wnt signaling pathway-related genes including axis inhibition protein 1 (AXIN1), β-catenin, lymphocyte enhancer-binding factor 1 (LEF1), cellular-myelocytomatosis (c-myc) and cyclin D1 on day 21 of osteoblasts were determined in Figure 4. Significant differences of mRNA/protein expression of these genes in different transfection groups of MSC-induced osteoblasts were found when compared with no transfection group (control group), all of these suppressed in analog + SCAI group and promoted in cs+ si-SCAI group (Figure 4, *P*<0.05). Thus, overexpression of SCAI attenuated the expression of these genes and may be involved in the activation of Wnt pathway by miR-409-3p.

### Blocking Wnt/β-catenin pathway attenuated the promotion of miR-409-3p on osteoblastic differentiation

**Figure 5:**
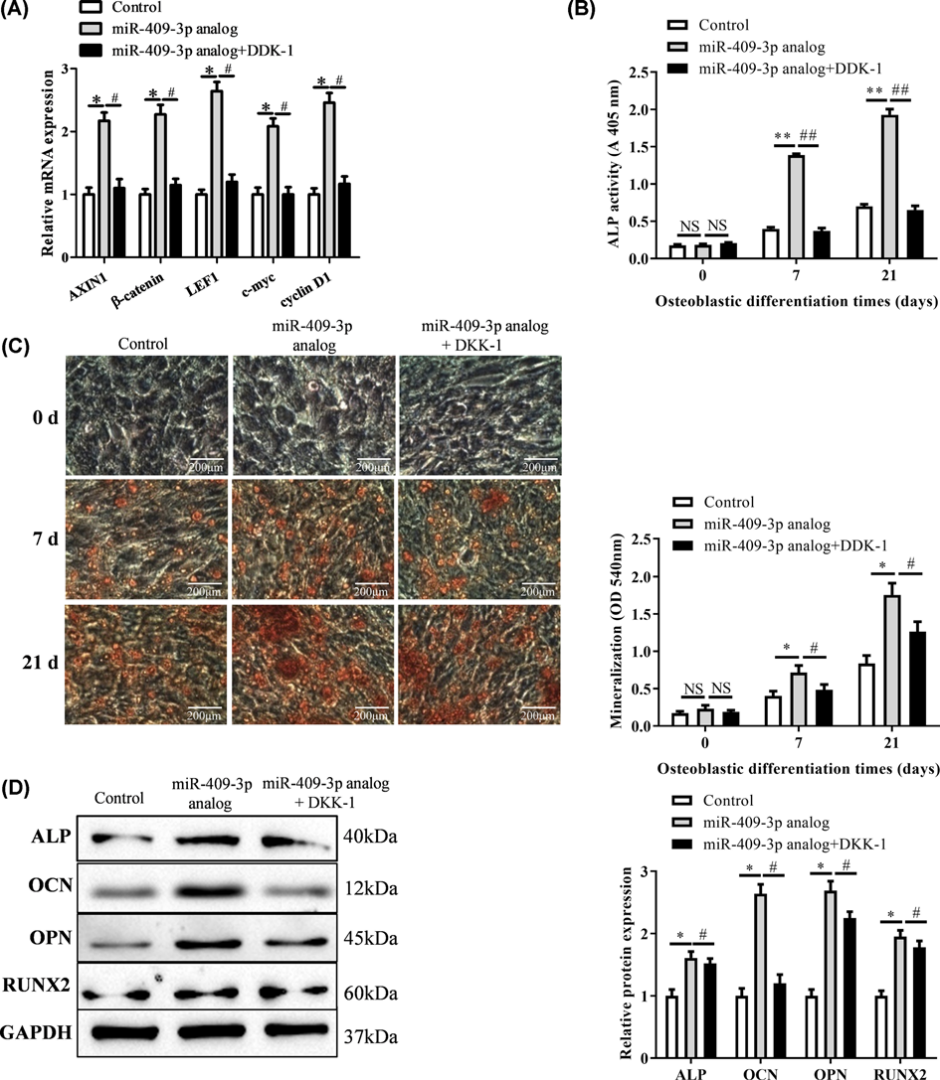
MiR-409-3p reduced osteoblastic differentiation by modulating Wnt/β-catenin signaling pathway After treatment with the Wnt signaling pathway inhibitor DKK-1 (**A**), the mRNA expression of AXINI, β-catenin, LEF1, c-myc, cyclin D1 was analyzed by RT-qPCR on day 21 of osteoblastic differentiation (vs. Control) (**B**) as well as the ALP activity (**C**), mineralization nodules (**D**), Western blotting analyzed osteogenesis-related gene expression (ALP, OCN, OPN, RUNX2) level in miR-409-3p analog and miR-409-3p analog + DKK-1 on day 21 of osteoblastic differentiation (vs. Control). *P<0.05, **P<0.01 (*, analog group; #, cs group). Abbreviation: NS, not significant.

In order to investigate the regulatory effect of Wnt signaling pathway on miR-409-3p induced osteoblast differentiation of MSCs, the Wnt inhibitor dickkopf-related protein 1 (DKK-1) was used [21]. The mRNA expressions of osteogenic markers were of significant difference in miR-409-3p analog when compared with the control group, but DKK-1 treatment almost reversed this increased trend (Figure 5A, *P*<0.05). Similar case was also reflected in ALP activity (Figure 5B, *P*<0.01). Next, we used ARS to investigate the mineralization nodules of miR-409-3p in MSCs induced osteoblasts after the 7th and 21at days of consecutive culture. The mineralized results of two groups were consistent with these above studies. At different times, the staining of mineralized nodules was remarkably reduced in the miR-409-3p analog + DKK-1 group when compared with the miR-409-3p analog group (Figure 5C, *P*<0.01). At last, the protein expression of the key markers for osteoblast differentiation-related genes, including ALP, OCN, OPN, RUNX2 [22], were also found to be markedly up-regulated in miR-409-3p analog group and down-regulated after DKK-1 treatment (Figure 5D, *P*<0.05). Overall, inhibition of Wnt signaling by DKK-1 treatment blocked miR-409-3p induced enhancement of osteoblastic differentiation of MSCs.

## Discussion

In the present study, we demonstrated experimentally that miR-409-3p showed a higher expression level during the osteogenic differentiation of MSCs. In addition, we found that the overexpression of miR-409-3p promoted the ability of osteogenic differentiation, but inhibited the expression of SCAI in osteogenic induction of MSCs. Moreover, overexpression of miR-409-3p promoted the activation of the Wnt/β-catenin signaling pathway by regulating *SCAI* and increased the expression of AXIN1, β-catenin, LEF1, c-myc and cyclin D1 [23], which leads to an increase in osteoblast differentiation. Furthermore, Wnt-specific inhibitor DKK-1 also interfered with the expression of miR-409-3p and affects osteogenic differentiation. Taken together, these results indicated that miR-409-3p may participate in the activation of the Wnt/β-catenin signaling pathway to increase osteoblastic differentiation through *SCAI*.

More and more studies have confirmed that miRNA can regulate gene expression and participate in a series of life activities, especially osteogenic differentiation. To investigate the role of miR-409-3p in osteoblasts differentiation, miR-409-3p analog and cs were first transfected into cells. Overexpression of miR-409-3p could enhance the osteoblastic differentiation of MSC-B cells, as featured with the increase of ALP activity and calcified nodules, which were recognized as osteoblast differentiation markers [24]. Furthermore, suppression of miR-409-3p showed the opposite effects. MiR-409-3p regulates several cellular behaviors [25]. In previous studies, low miR-409-3p expression was found in several tumors. For example, Chen et al*.* also found that miR-409-3p may be used as a tumor inhibitor in the progression of tongue squamous cell carcinoma via targeting radixin (*RDX*) [26]. Bai et al*.* also reported that miR-409-3p represses metastasis and invasion of colorectal cancer cell by targeting Grb2-associated binding protein (*GAB1*) expression [25]. Further on, according to the report of Cao et al*.*, low expression of miR-409-3p promoted breast cancer cell invasion [14]. However, in this study, miR-409-3p overexpression promoted the osteogenic differentiation of MSCs, which may have an important impact on resisting bone loss and bone degradation caused by cancer cell invasion.

SCAI, a possible tumor suppressor in regulating tumor cell invasion, was reported to make a difference in glioma cells by activating Wnt/β-catenin signaling [20]. This pathway is widely activated in a range of human solid tumors and plays a crucial role in tumor development by coordinating the dynamic balance of cell proliferation, invasion, metastasis and stem cell-like characteristics [27,28]. In addition, the Wnt signaling pathway is an important pathway that promotes the differentiation of osteoblasts and the absence of the multiple protein factors in this pathway can lead to bone defects [29]. Considering that *SCAI* is a direct target gene for miR-409-3p, a more in-depth understanding of whether *SCAI* regulates the osteogenic differentiation by interacting with Wnt/β-catenin signaling is well worth. Now, here we illustrated that overexpression of miR-409-3p reduced, while inhibition of miR-409-3p increased SCAI expression during the process of osteogenic differentiation. Moreover, overexpression of miR-409-3p suppressed the expression of Wnt pathway-related genes and the osteogenic differentiation by reducing SCAI. Recent studies show that SCAI, as the target gene of miR-1228, is down-regulated by miR-1228 and promoted the invasion and migration of osteosarcoma cells [30]. Similarly, the SCAI decreased in the study increased the osteogenic differentiation ability of MSCs.

Thus, to further investigate the role of Wnt signaling in miR-409-3p enhanced osteoblastic differentiation, we inhibited this signaling with the Wnt-specific inhibitor DKK-1. The results indicated that pretreatment with DKK-1 significantly reduced the osteogenic differentiation of MSC-B cells induced by miR-409-3p overexpression. Furthermore, Wnt/β-catenin silencing also reduced the formation of mineralization nodules, the ALP activity of osteoblasts and the protein expression of ALP, OCN, OPN, RUNX2. Thus, we demonstrated that miR-409-3p might play an important physiological effect in osteogenesis differentiation of MSCs through the Wnt/β-catenin signaling pathway *in vitro*. These results also proved a finding that Wnt signaling pathway was involved in the process of miR-409-3p induced osteoblasts differentiation.

Overall, the present study provides an essential operational link between miR-409-3p and osteogenesis differentiation. We demonstrated that overexpression of miR-409-3p promotes MSC-induced osteoblast differentiation and regulates osteoblast differentiation-related proteins to the higher level through the Wnt/β-catenin pathway by targeting *SCAI.* Therefore, as an osteogenesis regulator, miR-409-3p/*SCAI* interactions may exert a potential role in bone injury diseases treatments.

## Supplementary Material

Supplementary Figure S1

## Data Availability

All data generated or analyzed during the present study are included in this manuscript and supplementary materials.

## Abbreviations

ALP: alkaline phosphatase
ARS: Alizarin Red staining
AXIN1: axis inhibition protein 1
cs: complementary sequence
c-myc: cellular-myelocytomatosis
DKK-1: dickkopf-related protein 1
GFP: green fluorescent protein
HCO: human skull osteoblast
LEF1: lymphocyte enhancer-binding factor 1
miRNA: microRNA
miR-409-3p: microRNA-409-3p
MSC: mesenchymal stem cell
OCN: osteocalcin
OPN: osteopontin
RT-qPCR: reverse transcription-quantitative polymerase chain reaction
RUNX2: Runt-related transcription factor 2
SCAI: suppressor of cancer cell invasion
α-MEM: α-minimum essential medium
