# Supplementary material for: MicroRNA-409-3p promotes osteoblastic differentiation via activation of Wnt/β-catenin signaling pathway by targeting SCAI
Source: Biosci Rep. 2021 Jan 7;41(1):BSR20201902. doi: 10.1042/BSR20201902 (PMC7791548; doi:10.1042/BSR20201902)
Supplement: Supplementary Figure S1 [file BSR-2020-1902_supp.pdf]

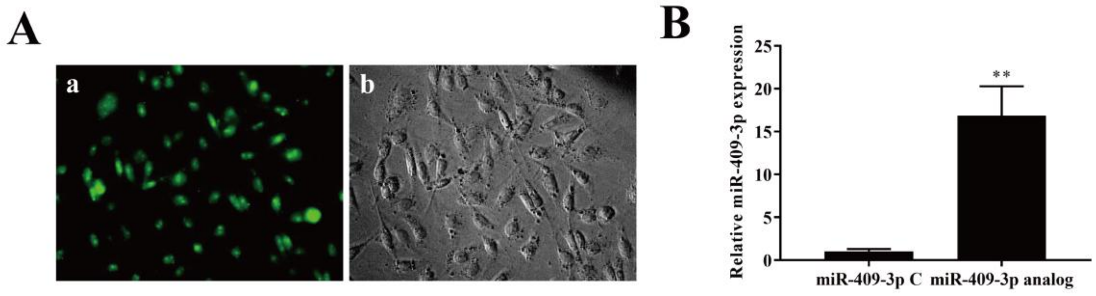

Supplementary Figure S1 (A) The transfection efficiency was evaluated by observing GFP fluorescence, a: green fluorescence; b: white light. (B) MiR-409-3p was highly increased after transfection of miR-409-3p analog compared to the cells transfected with miR-409-3p control.
